# Effects of propofol on renal ischemia/reperfusion injury in rats

**DOI:** 10.3892/etm.2013.1305

**Published:** 2013-09-18

**Authors:** SHUN YANG, WEI-PING CHOU, LING PEI

**Affiliations:** 1 Department of Anesthesiology, First University Hospital of China Medical University, Shenyang, Liaoning 110000;; 2 Department of Anesthesiology, Liaoning Cancer Hospital and Institute, Shenyang, Liaoning 110042, P.R. China

**Keywords:** propofol, ischemia/reperfusion injury, kidney, bone morphogenetic protein 2

## Abstract

Renal ischemia/reperfusion injury (IRI) is a major cause of acute renal failure. The aim of this study was to investigate whether propofol pretreatment in a rat model protects kidney tissue against IRI. Thirty-two Wistar rats were equally divided into four groups: a sham-operated group, untreated renal IRI group, and low-dose (5 mg/kg) and high-dose (10 mg/kg) propofol-treated groups which were treated with propofol prior to the induction of IRI. The rats were subjected to renal ischemia by bilateral clamping of the pedicles for 50 min, followed by reperfusion. The low-dose and high-dose propofol treatment groups were pretreated via femoral vein injection with a propofol suspension prior to the induction of ischemia/reperfusion. The untreated IRI group showed significantly higher serum creatinine (SCr), blood urea nitrogen (BUN), interleukin 6 (IL-6), IL-8, tumor necrosis factor-α (TNF-α), and malondialdehyde (MDA) levels compared with the sham-operated rats. Superoxide dismutase (SOD) levels were significantly reduced following IRI; however, they significantly increased following propofol administration. Bone morphogenetic protein 2 (BMP2) levels were significantly increased in the propofol-treated groups compared with the untreated IRI group. These results suggest that propofol reduces renal oxidative injury and facilitates repair following IRI. Propofol may play a protective role by regulating BMP2 expression in renal IRI.

## Introduction

Renal ischemia/reperfusion injury (IRI) is one of the underlying causes of acute renal failure (1,2). Inflammation substantially contributes to the pathogenesis of IRI with a central role for certain cells, adhesion molecules and cytokines (3). Neutrophils are inflammatory cells that are potent sources of reactive oxygen species (ROS), which are extensively generated during IRI and mediate cellular damage. Oxidative stress is caused by an imbalance between ROS production and anti-oxidant capacity. The formation of ROS and disturbances in the balance between oxidants and antioxidants play key roles in the mechanism by which renal IR causes tissue injury (4,5).

Propofol (2,6-diisopropylphenol) is a venous anesthetic extensively used in clinical practice and characterized by rapid induction and quick patient recovery from its effects. In addition, propofol is widely used as a sedative for intensive care patients. Propofol has a myocardial protective effect through a range of mechanisms, including oxygen free radical scavenging (6–8), blocking of the calcium channel by inhibiting the L-type calcium channel (9,10) and inhibiting neutrophil activation (11,12). The myocardial protective effect of propofol has been identified by a variety of methods using an IRI model (6–8,12,13). However, the potential benefits of propofol in ameliorating renal IRI and its mechanism of action remain unknown.

Bone morphogenetic protein 2 (BMP2) is a member of the multifunctional BMP family, which is part of the transforming growth factor β1 (TGF-β1) superfamily, originally identified as substances that induce bone and cartilage formation at ectopic extraskeletal sites *in vivo*. BMP2 in particular is heavily glycosylated and is involved in osteoblastogenesis (14). It not only plays a significant role in the regulation of cell proliferation and apoptosis, but also in the development and repair of various organs, including bone, nerve, heart and kidney (15,16).

In the current study, a regional renal IRI rat model was used to determine the effects of a clinically relevant propofol concentration during the peri-ischemic period of anesthesia. After 72 h of reperfusion, alterations in levels of the O_2_^−^ scavenger superoxide dismutase (SOD) and the lipid peroxidation by-product malondialdehyde (MDA) and the involvement of BMP2 in this process were investigated to determine whether propofol has a protective effect against renal IRI that involves these parameters of redox status.

## Materials and methods

### Animals

Male Wistar rats were obtained from the Animal Resource Center, Shenyang Pharmaceutical University (Shenyang, China). The rats were housed in cages with free access to water and food. Thirty-two male rats weighing 180–230 g were used. The Animal Ethics Committee of Liaoning Cancer Hospital and Institute approved this study and the experiments complied with established guidelines for animal care.

### Induction of kidney IRI

Rats were anesthetized with intraperitoneal injections of chloral hydrate (Tianjin Ruijinte Chemical Co., Ltd., Tianjin, China). Using a midline abdominal incision, the two renal pedicles were clamped for 50 min with microaneurysm clamps. During the period of ischemia, body temperature was maintained by placing rats on a 37°C heating pad. Following removal of the clamps, the kidneys were inspected for 1 min for restoration of blood flow, as noted by a return to their original color and then the abdomen was closed. Sham-operated rats received identical surgical procedures with the exception that microaneurysm clamps were not applied. To maintain fluid balance, all rats were supplemented with 5 ml saline administered via the femoral vein. Propofol was purchased from Qingyuan Jiabo Pharmaceutical Co. (Shenyang, China). Rats treated with propofol received identical surgical procedures with the exception that they were supplemented with 5 ml saline containing either 5 or 10 mg/kg propofol. The administration of saline or propofol was performed immediately prior to surgery. Rats were sacrificed 3 days after reperfusion. Blood was collected and kidney tissues were divided to be either snap frozen for subsequent mRNA extraction, fixed in 2% glutaraldehyde solution for electron microscopy or fixed in 10% neutral buffered formalin for paraffin embedding.

### Assessment of kidney function and oxidative stress

Serum creatinine (SCr) and blood urea nitrogen (BUN) were measured using the picric acid and diacetyl monoxime methods (17), respectively, in the Department of Biochemistry, Liaoning Cancer Hospital and Institute.

Serum levels of SOD and MDA were measured by chemical absorbance spectroscopy (18) in the Department of Biochemistry, Liaoning Cancer Hospital and Institute.

### Histological examination

Kidneys embedded in paraffin were sectioned at 3 *μ*m and stained with hematoxylin and eosin (H&E) by standard methods. Depending on the percentage of tubules in the corticomedullary junction that exhibited necrosis, loss of the brush border, cast formation or tubular dilatation, markers of tubular damage were scored from 0 to 5 (corresponding to none, ≤10, 11–25, 26–45, 46–75 and >76%, respectively) (19). Histological examination was performed by two blinded observers. At least ten high-power fields (HPFs; magnification, ×200) per section for each sample were examined.

### Electron microscopy

Following perfusion, kidneys were excised and immersed in fresh fixative (2.5% glutaraldehyde in 0.1 M sodium cacodylate buffer, pH 7.4) for 16 h at 4°C. For morphological studies, the tissue blocks were post-fixed with 1% osmium tetroxide and 0.8% potassium ferricyanide in 0.1 M cacodylate buffer, treated with aqueous 1% uranyl acetate, dehydrated in a graded ethanol series and embedded in PolyBed epoxy resin. Thin sections were cut, collected on 200-*μ*m mesh copper/rhodium grids, stained with sodium acetate and lead citrate and then observed at 60 kV with a transmission electron microscope (Hitachi, Tokyo, Japan).

### RNA extraction and quantitative polymerase chain reaction (PCR)

Total RNA was isolated from renal tissue using TRIzol (Invitrogen Life Technologies, Carlsbad, CA, USA). Equal amounts of RNA, measured by sfpectrophotometry and an RNA gel, were used for first-strand cDNA synthesis with Superscript II (Invitrogen Life Technologies) in a 20-*μ*l reaction. The cDNA product (1 *μ*l) was then subjected to reverse transcription-PCR (RT-PCR) with Taq polymerase (Boehringer Mannheim GmbH, Mannheim, Germany). Quantitative RT-PCR was performed using a Light Cycler system (Roche Diagnostics, Mannheim, Germany) and 2X SYBR Premix Ex Taq (Takara Bio Inc., Shiga, Japan) was used to detect PCR products. The comparative cycle threshold (Ct) method (2^−ΔΔCt^) was used to analyze relative changes in gene expression. β-actin (Actb) was used to normalize gene expression. The primer sequences were as follows: BMP-2, sense: 5′-ACGATGCCGCCATTTGTG-3′ and antisense: 5′-CGC CTCGCCTTCTTCAGT-3′, with the size of the PCR product being 349 bp; Actb, sense: 5′-GCCAACCGTGAAAAGATG-3′ and antisense: 5′-CCAGGATAGAGCCACCAAT-3′, with the size of the PCR producting being 701 bp.

### Kidney tissue protein extraction for cytokine measurements

Pre-chilled CelLytic MT reagent (Sigma-Aldrich, St. Louis, MO, USA) with a 1% protease inhibitor cocktail (Sigma-Aldrich) for use with mammalian tissue extracts was added to snap-frozen kidney tissue and then homogenized. The samples were incubated for 30 min at 4°C and centrifuged at 16,000 × g at 4°C for 15 min to pelletize the tissue debris. The supernatant was stored at −70°C. Protein concentrations were determined by a colorimetric protein assay (Bio-Rad, Hercules, CA, USA) using protein standards from Sigma-Aldrich.

### Enzyme-linked immunosorbent assay (ELISA)

Cytokines were measured in kidney homogenates using ELISA kits according to the manufacturer’s instructions. Kits for interleukin (IL)-6, IL-8 and tumor necrosis factor (TNF)-α were obtained from R&D Systems (Shanghai, China). Protein levels of cytokines were corrected for the total amounts of protein and the results were expressed in pg/ml.

### Western blotting

Aliquots (50 *μ*g) of kidney homogenates were separated on 10% polyacrylamide gels (Sigma-Aldrich) and transferred to a polyvinylidene fluoride membrane (PerkinElmer, San Jose, CA, USA). The membrane was blocked overnight in Western Blocker Solution (Sigma-Aldrich), incubated with anti-BMP2 antibody (Nventa Biopharmaceuticals Corporation, San Diego, CA, USA) in Western Blocker Solution for 1 h, washed, incubated with anti-mouse IgG conjugated with horseradish peroxidase (Sigma-Aldrich) and then washed. Positive bands were detected by chemiluminescence technology (Sigma-Aldrich) using the G:BOX gel documentation and analysis system (Syngene, Cambridge, UK). The membrane was also probed with anti-Actb antibody (Sigma-Aldrich) for Actb expression. The intensity of each band was quantified using Image J 1.32 software (National Institutes of Health, Bethesda, MD, USA).

### Statistical analyses

Results are expressed as mean ± standard deviation (SD). SPSS software (version 11.0; SPSS, Inc., Chicago, IL, USA) was used for all statistical analyses. Multiple groups were compared using one-way analysis of variance (ANOVA) with a post-hoc Bonferroni correction (GraphPad Prism 5.0; GraphPad Software). P<0.05 was considered to indicate a statistically significant difference.

## Results

### Rats are protected against kidney IRI by propofol injection

**Figure 1.**
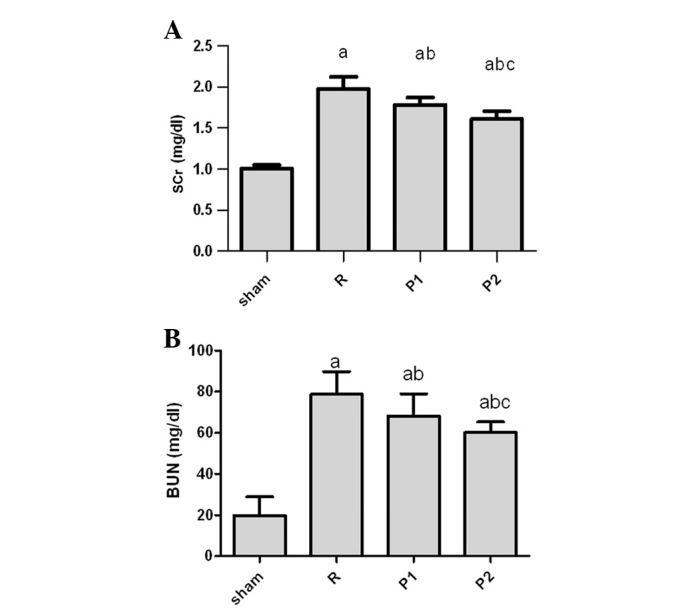
Propofol-treated rats were protected against renal ischemia/reperfusion injury (IRI) with significantly lower serum creatinine (SCr) and blood urea nitrogen (BUN) levels compared with untreated IRI controls following reperfusion. (A) SCr levels in the different groups. (B) Serum BUN levels in the different groups. Data are presented as mean ± standard deviation (SD); n=8 per group. S, sham-operated rats; R, operated rats; P1, rats treated with 5 mg/kg propofol; P2, rats treated with 10 mg/kg propofol. ^a^P<0.05 vs. S; ^b^P<0.05 vs. R; ^c^P<0.05 vs. P1.

As shown in Fig. 1A, IRI caused kidney dysfunction in untreated rats with a peak SCr of 1.92±0.44 mg/dl on day 3 after IRI as compared with 0.98±0.35 mg/dl in sham-operated rats. To determine the effects of propofol on kidney IRI, rats were injected with 5 or 10 mg/kg propofol (groups P1 or P2, respectively) in the IRI model. Rats treated with propofol were protected against the effects of ischemia, exhibiting significantly lower SCr and BUN levels than untreated rats. By contrast, there was an increase in SCr and BUN levels in rats treated with propofol on day 3, compared with sham rats (Fig. 1). The SCr and BUN levels in the P2 group were lower compared with those of the P1 group (Fig. 1).

**Figure 2.**
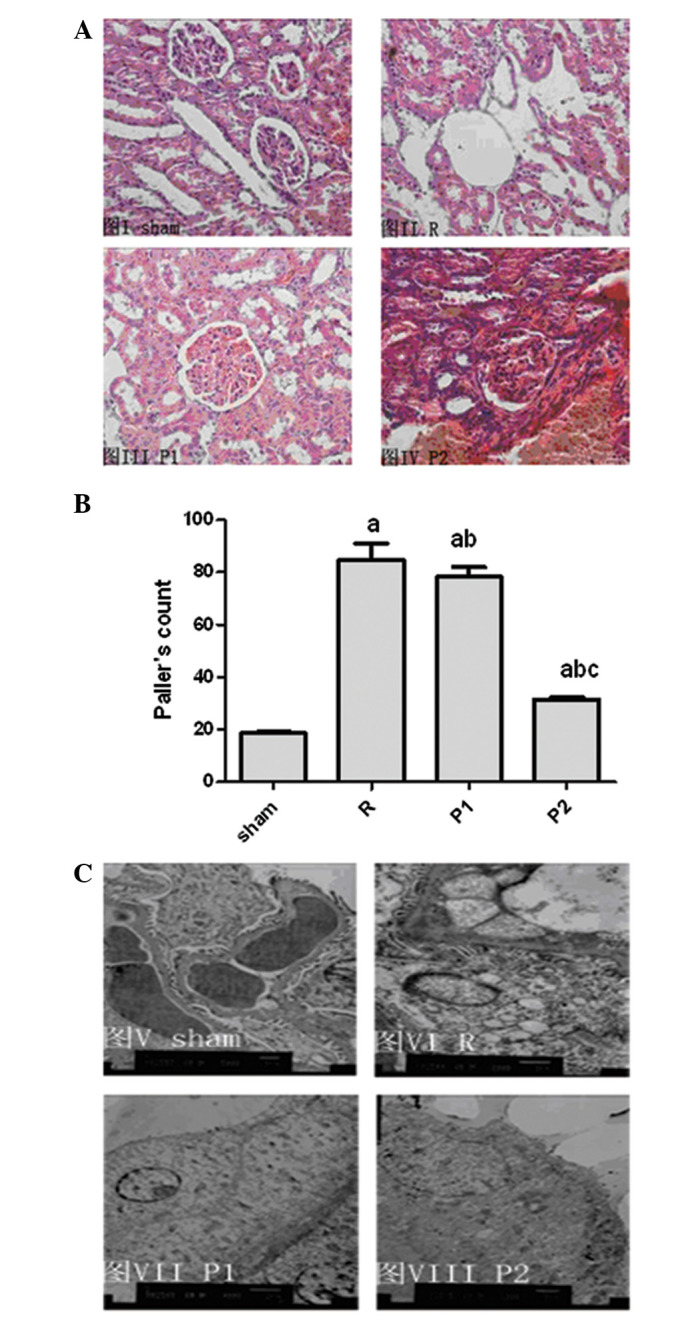
Tubular injury in propofol-treated ischemia/reperfusion injury (IRI) rats was significantly less compared with that observed in kidneys from untreated IRI rats. (A) Representative sections of the outer medulla from sham-operated, untreated IRI rats, rats treated with 5 mg/kg propofol and rats treated with 10 mg/kg propofol, three days after reperfusion (H&E; magnification, ×400). (B) Semiquantitative analysis of tubular damage in the kidneys of sham-operated rats, operated rats, rats treated with 5 mg/kg propofol and rats treated with 10 mg/kg propofol following reperfusion. Data are presented as mean ± standard deviation (SD); n=8 per group. ^a^P<0.05 vs. S; ^b^P<0.05 vs. R; ^c^P<0.05 vs. P1. (C) Representative electron micrograph sections from sham-operated rats, operated rats, rats treated with 5 mg/kg propofol and rats treated with 10 mg/kg propofol three days after reperfusion. Original magnification, ×7,200. S, sham-operated rats; R, operated rats; P1, rats treated with 5 mg/kg propofol; P2, rats treated with 10 mg/kg propofol.

The functional data correlated with histological kidney tubular damage. Severe tubular damage was observed in untreated IRI rats with no propofol treatment, as shown by widespread tubular necrosis, loss of the brush border, cast formation and tubular dilatation at the corticomedullary junction, whereas IRI rats with propofol treatment demonstrated significantly less tubular damage (Fig. 2A). Sham-operated rats incurred no tubular injury. A blind review of specimens from untreated IRI rats revealed greater tubular injury in their kidneys. The mean histological score for the kidneys of the propofol-treated (10 mg/kg) rats was 32.8±0.6% compared with 85.3±6.1% (P<0.01; Fig. 2B) for the untreated IRI control. Tubular injury was attenuated as the propofol dosage increased (Fig. 2B).

Morphological studies using electron microscopy demonstrated a certain degree of heterogeneous loss of brush border, bleb formation, cytoplasmic vacuolization, cellular necrosis, mitochondrial loss or disappearance, chromatin condensation and aggregation at the periphery of nuclei and nuclear fragmentation, and tubular luminal debris and obstruction in untreated kidneys. Damage was markedly reduced in propofol-treated kidneys (Fig. 2C).

### Oxygen free radical scavenger SOD levels are increased in propofol-treated rats

**Figure 3.**
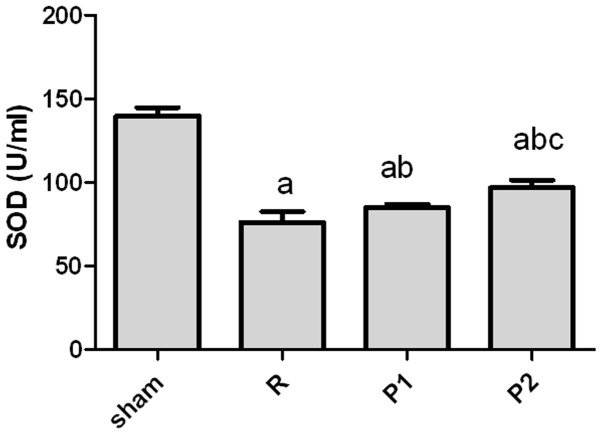
Superoxide dismutase (SOD) activity following ischemia/reperfusion injury (IRI). Data are presented as mean ± standard deviation (SD); n=8 per group. ^a^P<0.05 vs. S; ^b^P<0.05 vs. R; ^c^P<0.05 vs. P1. S, sham-operated rats; R, operated rats; P1, rats treated with 5 mg/kg propofol; P2, rats treated with 10 mg/kg propofol.

Normally, tissues contain enough endogenous scavengers to protect against damage induced by oxygen free radicals (OFRs). The levels of SOD, one such scavenger, were measured following IRI. The SOD levels were significantly reduced following IRI compared with those in sham-operated controls (Fig. 3). Propofol-treated rats had significantly higher SOD levels compared with untreated rats following IRI (Fig. 3). Moreover, rats treated with 10 mg/kg propofol had significantly higher SOD levels compared with those treated with 5 mg/kg propofol (Fig. 3).

### Lipid peroxidation by OFRs is reduced in propofol-treated rats

**Figure 4.**
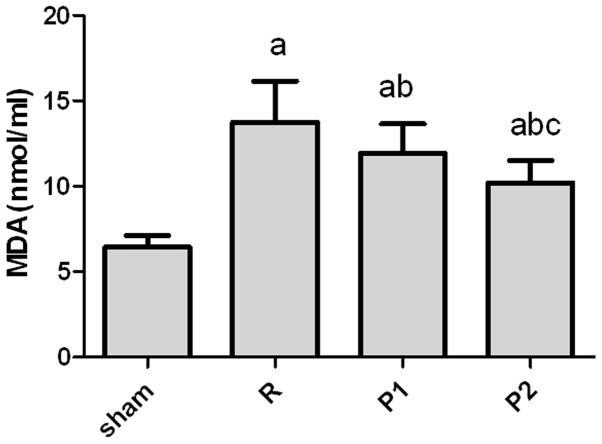
Lipid peroxidation following ischemia/reperfusion injury (IRI). Data are presented as mean ± standard deviation (SD); n=8 per group. ^a^P<0.05 vs. S; ^b^P< 0.05 vs. R; ^c^P<0.05 vs. P1. S, sham-operated rats; R, operated rats; P1, rats treated with 5 mg/kg propofol; P2, rats treated with 10 mg/kg propofol; MDA, malondialdehyde.

The effect of IRI on lipid peroxidation was evaluated in the rats. The lipid peroxidation by-product MDA was measured as a marker for membrane lipid peroxidation. IRI resulted in an increase in the whole kidney MDA levels of operated groups compared with that of the sham-operated group (Fig. 4).

Propofol-treated rats demonstrated less lipid peroxidation with significantly lower MDA levels compared with the untreated IRI controls. Rats treated with 10 mg/kg propofol had significantly lowere levels of MDA compared with those treated with 5 mg/kg propofol (Fig. 4).

### Propofol attenuates pro-inflammatory cytokine expression in the kidney during IRI

**Figure 5.**
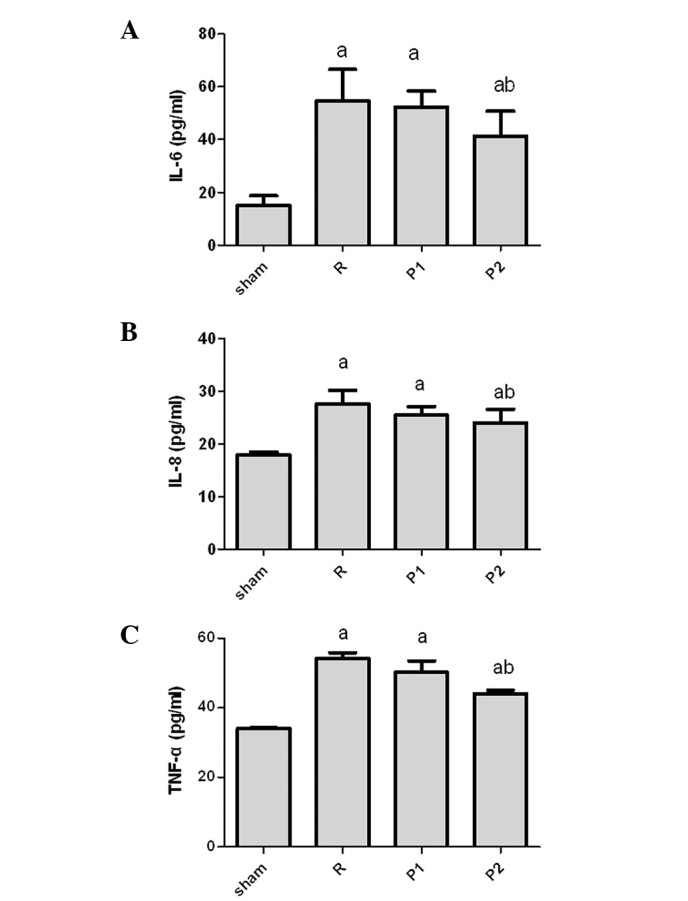
Effects of propofol on cytokine levels following ischemia/reperfusion injury (IRI). Levels of pro-inflammatory cytokines [interleukin (IL)-6, IL-8, and tumor necrosis factor (TNF)-α] in the kidney were significantly reduced in P1 (5 mg/kg) and P2 (10 mg/kg) rats compared with controls following reperfusion. Data are presented as mean ± standard deviation (SD); n=8 per group. ^a^P<0.05 vs. S; ^b^P<0.05 vs. R. S, sham-operated rats; R, operated rats; P1, rats treated with 5 mg/kg propofol; P2, rats treated with 10 mg/kg propofol.

To further determine the effects of propofol in the IRI kidney model, we examined the expression of cytokines (Fig. 5). IL-6, IL-8 and TNF-α levels were significantly increased in the IRI kidney compared with those in sham-operated controls. Cytokine levels increased in rats treated with 10 mg/kg propofol; however, the extent of the increase was much less than that in untreated IRI rats. No significant differences were identified between the two groups treated with different doses of propofol (Fig. 5).

### Role of BMP2 in the kidney during IRI

**Figure 6.**
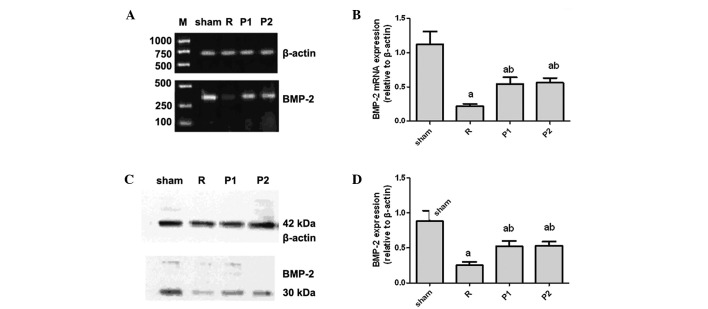
Bone morphogenetic protein 2 (BMP2) expression following ischemia/reperfusion injury (IRI). (A) BMP2 mRNA expression. (B) Quantitative reverse transcription-polymerase chain reaction (RT-PCR) analysis of BMP2 mRNA levels in the different groups. (C) Western blot analyses of BMP2 protein expression in the different groups. Representative results are shown. (D) The relative quantitation of BMP2 protein. S, sham-operated rats; R, operated rats; P1, rats treated with 5 mg/kg propofol; P2, rats treated with 10 mg/kg propofol. ^a^P<0.05 vs. S; ^b^P<0.05 vs. R.

To determine the role of BMP2 in our model, we measured mRNA expression levels of BMP2 in the kidney by real-time quantitative PCR. The level of BMP2 mRNA was significantly reduced following IRI compared with that in sham-operated controls (Fig. 6A and B). Sham-operated kidneys expressed abundant BMP2, whereas BMP2 mRNA expression decreased markedly in kidneys following IRI (Fig. 6A and B). By contrast, the reduction in mRNA expression for BMP2 was less in propofol-treated kidneys than in untreated kidneys (Fig. 6A and B).

Consistent with the real-time PCR data, western blots revealed that IRI induced a significant reduction in BMP2 expression in the kidneys of untreated IRI rats compared with that in sham-operated controls (Fig. 6C and D). Downregulation of BMP2 expression following IRI was greatly attenuated in the propofol-treated rats.

## Discussion

Previous experimental data suggests that IRI rapidly induces the formation of OFRs, while SOD levels are reduced in the kidneys following IRI (20,21). In the present study we identified that IRI resulted in increased production of the lipid peroxidation by-product MDA in homogenates of whole kidneys. By contrast, propofol pretreatment reduced MDA levels, increased SOD levels and improved renal dysfunction following IRI. We identified that rats pretreated with propofol were protected from kidney dysfunction and histological damage. Protection was associated with a reduction in pro-inflammatory cytokine generation and a concomitant increase in BMP2 expression.

The most common cause of acute renal failure is renal ischemia, which causes functional impairment through a combination of renal vasoconstriction, tubular obstruction, tubular back-leakage of glomerular filtrate and reduced glomerular permeability (22). IRI results in the activation of multiple cell injury pathways that contribute to organ dysfunction, including those resulting in the production of OFRs (23).

OFRs are considered to cause cellular injury by attacking membranes through the peroxidation of polyunsaturated fatty acids (24,25) during IRI. This lipid peroxidation results in increased membrane permeability in cells, mitochondria and lysosomes. Peroxidative injury of erythrocyte membranes has been reported to increase passive K^+^ permeability with a loss of intracellular K^+^(26). Increased permeability of renal tubular cell membranes may lead to a loss of transport functions, whereas increased permeability of mitochondrial membranes impairs oxidative phosphorylation. Increased lysosomal permeability may result in the leakage of hydrolytic enzymes and acceleration of cellular degradation. In the current study, we identified that IRI increased the production of the lipid peroxidation by-product MDA in the homogenates of whole rat kidneys.

Propofol has been reported to modulate IRI, suggesting its potential for organ protection during surgery involving abdominal aortic clamping and possibly as a means for improving patient outcome (27). Propofol is a lipophilic hypnotic drug with proven antioxidant activity in *in vitro* and *in vivo* studies. This results in part from its chemical structure, which is similar to the natural antioxidant vitamin E (5,14). Propofol has been demonstrated to act as a scavenger of OFRs, reducing lipid peroxidation in the liver, kidney, heart and lung (28). It was reported that in an *in vivo* experimental model of reversible renal IRI, propofol anesthesia was associated with diminished neutrophil infiltration, and reductions in plasma pro-inflammatory cytokine levels, production of OFRs, lipid peroxidation and inducible nitric oxide synthase activity (29). Results from the study by Yuzbasioglu *et al* (30) demonstrated that IRI was significantly reduced in the presence of propofol and that the protective effects of propofol may be due to its antioxidant properties. Results from the study by Yuzer *et al* (31) demonstrated that IRI was significantly reduced in the presence of propofol and thiopental. The authors attributed the protective effects of these drugs to their antioxidant properties. In the current study, propofol pretreatment was observed to reduce lipid peroxidation, increase SOD levels and suppress the production of inflammatory cytokines.

BMPs are members of the TGF-β1 superfamily and play important roles in diverse cell types. Vascular endothelial and smooth muscle cells express BMP receptors and secrete BMPs (30–32). Among them, BMP2 has been shown to regulate a host of cellular functions (33), including cardiovascular development, angiogenesis, neovascularization in tumors, vascular calcification and smooth muscle cell chemotaxis, in response to vascular injury (34,35). It is considered that BMP signaling exerts important vasoprotective effects controlling the balance between proliferation and activation of apoptosis in endothelial and smooth muscle cells (36).

BMP2 is considered to signal primarily by activating the mothers against decapentaplegic (SMAD) and mitogen-activated protein kinase (MAPK) pathways (37), although evidence suggests that BMP2 may also activate nuclear factor κ-light-chain-enhancer of activated B cells (NF-κB) (38). The activation of BMP signaling, either by overexpression of BMP2 in vascular cells or administration of recombinant BMPs, results in endothelial dysfunction, oxidative stress and enhanced monocyte adhesiveness to the endothelium (39). BMP2 is selectively expressed by late outgrowth endothelial progenitor cells and plays a role in neoangiogenesis (40). Endothelial colony-forming cells (ECFCs) express BMP2 morphogens. BMP2 may be used as a marker of immaturity, the ECFC lineage and finally as an angiogenic marker during ECFC commitment and expansion. BMP-positive endothelial precursors correspond to ECFCs, responsible for neovascularization, whereas BMP-negative endothelial precursors correspond to proangiogenic hematopoietic progenitor cells (41). A previous study demonstrated that BMP2 is downregulated following IRI, which may contribute to an imbalance between cell proliferation and apoptosis, thereby causing renal injury (42). In the present study, propofol pretreatment was observed to promote BMP2 expression following IRI and may have contributed to neoangiogenesis, which may partly explain its renal protective effect. Future studies are required to elucidate the mechanism by which propofol regulates the expression of BMP2.

In conclusion, regulation of BMP2 levels may be an important mechanism for maintenance of cellular homeostasis. Propofol pretreatment exerts a protective effect in rats in an IRI model, which is partly correlated with upregulation of BMP2. This study may open new avenues of investigation into the antioxidant effects of propofol. An understanding of the mechanisms of action of propofol in IRI may introduce new therapeutic approaches not presently available.
